# Microbial Infections and Antimicrobial Use in Neonates and Infants

**DOI:** 10.3390/tropicalmed10030080

**Published:** 2025-03-18

**Authors:** Chryssoula Tzialla

**Affiliations:** Neonatal and Pediatric Unit, Polo Ospedaliero Oltrepò, ASST Pavia, 27058 Voghera, Italy; chryssoula.tzialla@unipv.it

## 1. Introduction

Infectious diseases in infants are a major cause of morbidity and mortality during the first 28 days of life, accounting for approximately 33% of neonatal deaths and 50% of deaths in children under the age of five [1]. Moreover, infants who survive serious bacterial infections (SBI) face a risk of long-term disability [2], and SBI contributes to approximately 3% of all disability-adjusted life years in global burden [3].

Neonatal infections are categorized as early-onset or late-onset. Early-onset infections usually arise from vertical transmission, including transplacental spread in late pregnancy or ascending infection during childbirth. In contrast, late-onset infections are primarily associated with nosocomial transmission and are acquired horizontally after birth. According to the World Health Organization (WHO), neonatal infections are primarily bacterial in origin, presenting as pneumonia, sepsis, and meningitis, and are responsible for over 550,000 neonatal deaths annually [3].

The risk of sepsis is particularly high during the neonatal period, which represents the time of maximum lifetime risk. According to a 2022 study, globally, an estimated 1.3 to 3.9 million young infants experience sepsis annually, resulting in approximately 400,000 to 700,000 sepsis-related deaths [4]. However, global incidence and mortality rates remain uncertain due to a lack of accurate data and surveillance systems, particularly in low- and middle-income countries. Infants with low birth weight, very low birth weight (VLBW), and those born preterm admitted to neonatal intensive care units (NICUs) are especially vulnerable to hospital-acquired infections. This increased risk is attributed to the frequent use of invasive medical devices (e.g., central lines), prolonged hospital stays, and associated comorbidities [5].

In NICUs, more than half of hospital-acquired infections are sepsis-related, with an estimated mortality rate 5.5% higher in affected infants compared to those not affected [6]. Healthcare-associated sepsis (HAS) is also linked to longer hospital stays and higher rates of antimicrobial resistance compared to community-acquired sepsis. Importantly, many cases of HAS could be prevented with proper infection control measures [7].

Early detection and treatment of neonatal infections are crucial in reducing mortality. Current diagnostic methods rely on blood, cerebrospinal fluid, and urine culture in suspected cases of conditions such as bacteremia, meningitis, and pneumonia [8]. Although microbiological culture remains the gold standard, it is time-consuming and prone to false negatives. Notably, microbial culture detection rates are as low as 3–10% in early-onset sepsis and 16.7–33% in late-onset sepsis [1]. Furthermore, clinical signs in neonates are often nonspecific and overlap with other clinical conditions [8]

Molecular techniques, such as polymerase chain reaction (PCR) and 16S rRNA gene sequencing, have shown promise in improving microbial detection. The study by Agudelo-Pérez et al. [Contribution 1] investigated the role of PCR targeting the variable region V4 of the 16S ribosomal gene (16S rDNA) and Sanger sequencing for bacterial identification in preterm infants with suspected early-onset neonatal sepsis. The study demonstrated that molecular testing allowed the detection of microbial species in 67.8% of samples from preterm neonates with suspected early-onset sepsis (EOS) but negative blood cultures. This is particularly significant, as approximately 95% of preterm infants receiving antibiotics for suspected early-onset neonatal sepsis lack microbiological confirmation of infection, resulting in unnecessary antibiotic use and associated health risks [9,10]. Therefore, developing precise, unbiased, and rapid diagnostic techniques is essential for the effective management of neonatal infections. As De Rose et al. [Contribution 2] highlight in their review on the current challenges and future perspectives in the diagnosis and management of neonatal bacterial sepsis, in the near future artificial intelligence has the potential to aid in developing treatment algorithms and advancing personalized medicine.

In parallel with neonatal sepsis, antimicrobial resistance (AMR) represents a growing threat. The diminishing efficacy of antibiotics risks a return to a pre-antibiotic era, where infections become increasingly difficult to treat. According to the WHO, AMR is one of the top 10 global health threats. In 2019, approximately 5 million deaths were associated with AMR, with projections estimating up to 10 million annual deaths by 2050, surpassing cancer-related mortality [11,12].

AMR spreads rapidly across continents, affecting humans, animals, and goods. Hospitals, in particular, serve as hotspots for the transmission of antibiotic-resistant microorganisms, which can spread from patient to patient or through environmental contamination. The primary drivers of AMR include excessive and inappropriate antibiotic use in both human medicine and livestock. Alarmingly, antibiotic consumption is expected to increase by 200% by 2030, exacerbating the resistance crisis [13].

In neonatal care, antibiotic use remains a significant challenge. Neonates, particularly pre-term infants, are at high risk of infections and frequently receive empirical antibiotic therapy. Approximately 14% of late pre-term and full-term neonates and up to 90% of extremely low-birth weight infants receive empirical antibiotics immediately after birth, despite the fact that culture-confirmed EOS occurs in only a small percentage of cases [14,15]. Alarmingly, early and prolonged exposure to antibiotics may lead to dysbiosis, contribute to the development of antimicrobial resistance, and contribute to short-term and potential long-term health consequences [15,16,17].

The rapid emergence of antibiotic-resistant Gram-negative bacteria is particularly concerning in neonatology. Strains such as β-lactamase-producing Enterobacteriaceae and carbapenem-resistant bacteria are making neonatal infections increasingly difficult to treat [18]. The lack of new antibiotics and the restricted use of existing ones further intensify the crisis.

To combat AMR, a global, multi-sectoral approach is needed, involving healthcare, veterinary, agricultural, and policy-making sectors. Prevention strategies, such as improved hand hygiene, infection control protocols, and antibiotic use monitoring, are essential. Hospitals should implement “Antibiotic Stewardship” (AS) programs to optimize antibiotic prescribing practices and minimize the selection pressure driving resistance [19].

Although research on AS programs in neonates is limited, evidence indicates that careful antibiotic use benefits VLBW infants, especially during the first week of life [15]. Quality improvement initiatives like minimizing unnecessary antibiotic exposure in preterm infants without perinatal EOS risk factors have proven effective in safely reducing antibiotic overuse in the short term [20]. While the effectiveness of AS programs has been demonstrated, data on the long-term sustainability of AS interventions —particularly in infants with lower gestational ages and birth weight—remain scarce.

The study by Zini et al. [Contribution 3] highlights that AS is not only feasible in preterm VLBW infants but also can safely reduce antibiotic use by shortening antibiotic courses in uninfected neonates. Additionally, the study demonstrates that AS practices can be effectively sustained over time through clinical audits and daily discussions among healthcare staff.

Similarly, De Rose et al. [Contribution 4] emphasized two key aspects of managing late-onset sepsis: when to initiate and when to discontinue antibiotic therapy. Clinical symptoms trigger diagnostic work-up, supported by biomarkers such as C-reactive protein, procalcitonin (PCT), and presepsin, to guide the decision to start antibiotic treatment. Current protocols recommend discontinuing antibiotics after 36–48 h if blood cultures are negative and the infant is clinically stable. However, standardized, biomarker-guided antibiotic stewardship protocols are essential to optimize treatment decisions. To date, PCT is the marker with the most robust evidence supporting its effectiveness in this context. Ultimately, antimicrobial stewardship is a responsibility shared by all healthcare professionals.

In conclusion, neonatal infections and antimicrobial resistance present complex and evolving challenges. The vulnerability of neonates to infections, coupled with the rise in AMR, underscores the urgent need for continued research, improved diagnostics, and stringent infection control measures. Through collaboration among healthcare providers, researchers, and policymakers, the burden of neonatal infections can be reduced, safeguarding the health of future generations. Implementing effective antimicrobial stewardship practices, alongside novel therapeutic approaches, holds great promise for mitigating the impact of infections and resistance in this critical population.

## Abbreviations

The following abbreviations are used in this manuscript:

AMR	Antimicrobial resistance
AS	Antibiotic stewardship
EOS	Early-onset sepsis
HAS	Healthcare-associated sepsis
PCR	Polymerase chain reaction
PCT	Procalcitonin
NICU	Neonatal intensive care
VLBW	Very low birth weight
WHO	World Health Organization
